# Skewed Inactivation of X Chromosome: A Cause of Hemophilia Manifestation in Carrier Females

**DOI:** 10.7759/cureus.11216

**Published:** 2020-10-28

**Authors:** Hafiz Muhammad Hassan Shoukat, Ghulam Ghous, Zahid Ijaz Tarar, Muhammad Mohsin Shoukat, Namra Ajmal

**Affiliations:** 1 Internal Medicine, Premier Health/Wright State University, Dayton, USA; 2 Internal Medicine, University of Missouri, Columbia, USA; 3 Pediatric Medicine, Children's Hospital, Lahore, PAK; 4 Pathology, King Edward Medical University, Mayo Hospital, Lahore, PAK

**Keywords:** hemophilia, lyonization, clotting disorder, bar body

## Abstract

Hemophilia is an X-linked recessive hereditary disorder that classically affects males due to the presence of only one X chromosome in males. Females are usually carriers due to the presence of counterpart X chromosome, but many times manifestations of hemophilia are seen in heterozygous carrier females. This is a result of skewed lionization, in which more normal X chromosomes are converted to bar body, and more abnormal chromosomes remain active in body cells, causing the dominant manifestation of the disease. The severity of manifestations is directly proportional to the level of the clotting factor in the blood. The disease can be severe enough to cause life-threatening bleeding, especially during delivery. Physicians usually reluctant to assume hemophilia in the differential diagnosis of the bleeding disorders in women but manifesting carrier females with hemophilia are not uncommon. Our review of the literature will give an opportunity to understand this issue more precisely as well as will discuss the disease manifestations and its updated management.

## Introduction and background

Hemophilia is the most common hereditary [1] clotting disorder characterized by impaired coagulation leading to bleeding diathesis depending on the severity of the case. There are three types of heritable hemophilia, classified as type A, B, and C, all caused by the mutations in genes responsible for clotting factor (F) VIII, IX, and XI respectively. Genes for FVIII and FIX are located on chromosome X in the human genome, whereas the gene responsible for FXI is on chromosome 4. Hemophilia A (FVIII deficiency) makes up to 80-90% of the total disease burden, hemophilia B makes up to 10-20%, and hemophilia C is mainly concentrated in the Jewish population of Ashkenazi descent [2,3].

## Review

Mode of transmission

**Figure 1:**
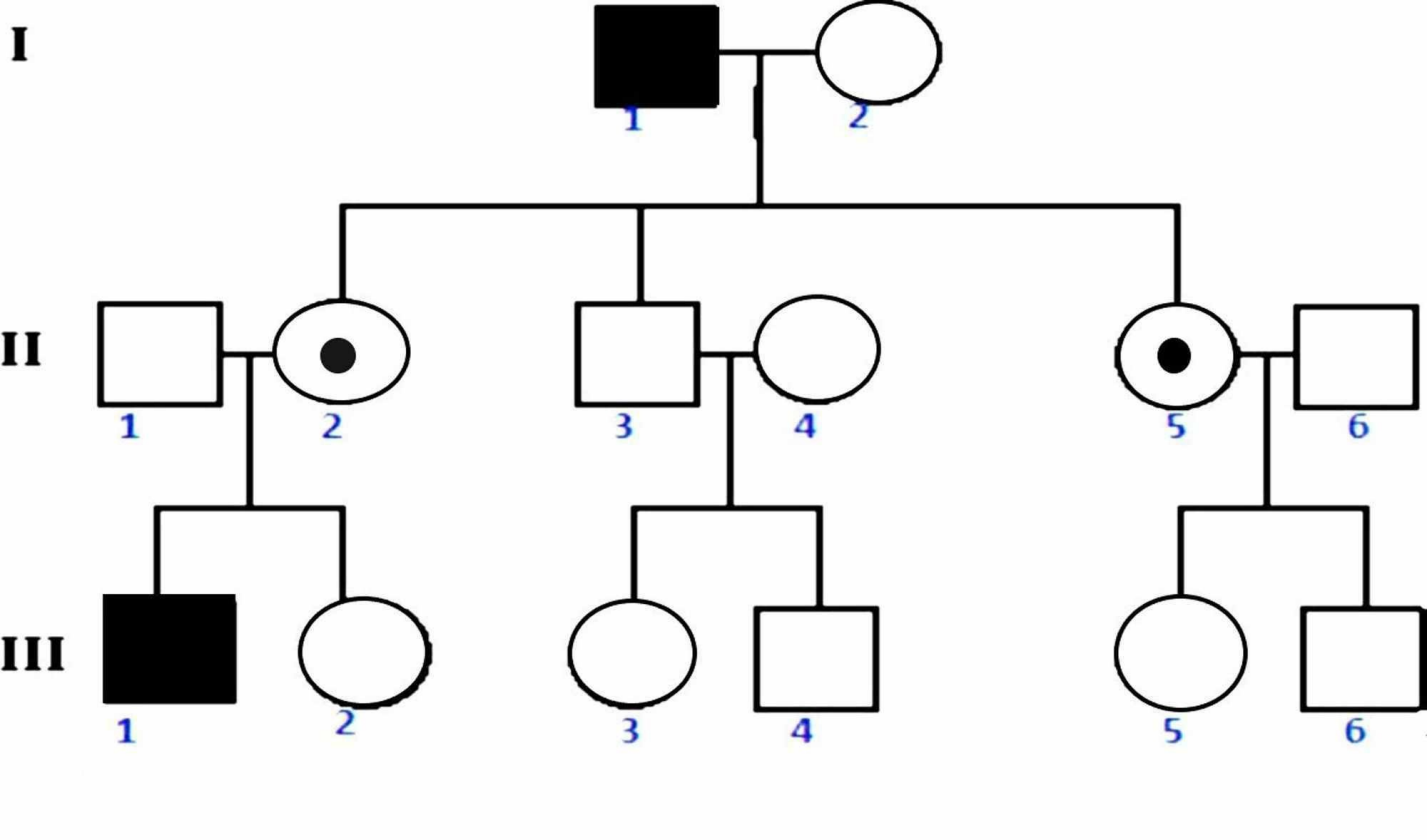
Pedigree: X-linked recessive inheritance Rectangle: male; circle: female; filled rectangle: affected male; dotted circle: affected female. Female 2 (II) passed hemophiliac X to her son 1 (III).

Although de novo mutations are frequently observed, making up to 30% of total hemophilia [1], the mode of inheritance of both hemophilia A and B is X-linked recessive inheritance. Hemophilia C is a non-X-linked disorder with an autosomal mode of inheritance. Both hemophilia A and B are transmitted in a zigzag pattern, from mothers to sons and from fathers to daughters (Figure 1) [4,5]:

1) Affected male 1 can transmit mutated X chromosome to daughters only.

2) Carrier female 2 can transmit the mutated X chromosome to half of her daughters (carrier) and half sons (affected).

3) Affected homozygous female, which is an extremely rare case, will transmit the mutated gene to 100% of her daughters (carrier) and 100% of her sons (affected).

Discussion

**Figure 2:**
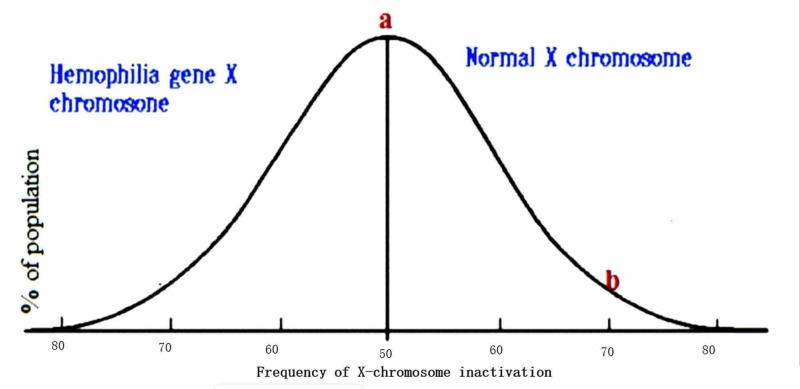
X chromosome inactivation pattern in hemophilia carrier females

Lyonization is a process by which one of two X chromosomes in a female randomly becomes inactivated, forming a bar body early during fetal life; the process of inactivation is just by random selection. The inactive X chromosome becomes a part of heterochromatin as it is transcriptionally inactive due to its tight packing. As nearly all females have two X chromosomes, the process of lyonization prevents them from having twice as many X chromosome gene products as males, who only possess a single copy of the X chromosome. Although this inactivation is random but permanent in the cell, the same chromosome remains inactive throughout that cell's progeny. On average, 50% of the maternal X chromosome and 50% of paternal X chromosomes become inactivated in all the body's somatic cells by random selection. It is depicted by a normal distribution curve in the general population (point a) (Figure 2). But as on both extremes, distribution becomes skewed, and one type of chromosomes becomes more inactivated in some populations of females [6-10]. The risk of disease manifestations increases as more normal X chromosomes become inactive as depicted by point b. However, there is a poor correlation between plasma clotting factor level and X chromosome inactivation pattern in some studies [2,11].

Other possible causes of hemophilia manifestation in females are homozygous hemophilia status, Turner syndrome in hemophilia carriers, and acquired hemophilia [12]. Severe deficiency of vitamin K can cause hemophilia-like manifestations as well as von Willebrand disease, which can mimic type A hemophilia.

Classical bleeding in hemophilia is mainly deep, and internal organ bleeds after minor trauma. Bleeding in joints and internal organs are serious hemophilia complications. Hemophilia types cannot be distinguished on a clinical basis. All have the same presentations with variability in severity. Patients with milder disease usually remain asymptomatic until undergoing surgery or major trauma and may remain undiagnosed for many years, especially if they do not undergo circumcision. Moderate-to-severe diseases have more frequent bleeding problems. Deep bleeds are more common than superficial skin bruises in hemophilia. Hemarthrosis causes joint pain, swelling, and, ultimately, joint contracture, limiting joint mobility. Muscular hematomas may acutely press over nerves, leading to parenthesis distally and chronically, causing muscle contracture. Deep bleeding in the brain is usually grievous, leading to severe headache, neurological deficit, and increased intracranial pressure. Other common manifestations include extensive ecchymosis, gastrointestinal, urinary tract bleeds, and epistaxis without any known cause. Hemophilia C does not have a specific bleeding pattern and is usually a mild disease without hemarthrosis, hematoma, and brain bleed. In manifesting females, the bleeding tendency leads to prolonging and heavy menstrual bleeding (menorrhagia), and the risk of bleeding further increases with uterine abnormalities such as polyps and fibroids. Manifesting females also more frequently experience mid-cycle abdominal or pelvic pain (mittelschmerz) during ovulation with internal bleeding that sometimes can be extensive and severe.

In hemophilic children, the initial symptom is usually prolonged bleeding after circumcision and vaccination (from injection site). Vacuum extractor or forceps use during delivery can cause cephalic hematoma or large ecchymosis. Initial laboratory clue to hemophilia is prolonged aPTT (activated partial thromboplastin time) values with normal PT (prothrombin time), bleeding time, and platelet count. Diagnosis is confirmed by measuring the clotting factor (FVIII, FIX) activity in plasma. The average age of diagnosis of hemophilia is nine months of age, with almost all moderate-to-severe cases diagnosed by the age of two.

Some studies suggest no correlation between X chromosome inactivation pattern and plasma concentration of clotting factors [2,11], but the severity of disease directly correlates with the levels of clotting factor in the blood. Mild disease is categorized when blood clotting factor activity is 5-50% of normal, moderate disease with activity 1-5%, and severe form with <1% of normal factor activity (Table 1) [3].

**Table 1:** Hemophilia classification based on the severity

Level	% Factor activity in blood	Average age at diagnosis	Average frequency bleeding episodes
Normal	50-150%		No abnormal bleeding
Mild	6-50%	Late diagnosis (usually in adulthood)	Mostly remain asymptomatic, bleeding with major trauma/surgery
Moderate	1-5%	1-2 years of age	Bleeding with minor trauma (4-6 bleeds/year)
Severe	<1%	Early diagnosis < 1 year of age	Spontaneous frequent bleeding episodes (2-4 bleeds/month)

Around one-third of all carrier females have factor activity below 50% of normal and exhibit mild-to-moderate manifestations of hemophilia. All carrier females should be evaluated for factor activity and precautionary measures should be advised, such as the following:

1) Avoidance of contact supports.

2) Avoid taking any medicine (aspirin etc.) without physician advice.

3) Evaluation for menorrhagia and subsequent anemia; if present iron and folic acid supplements should be advised.

Prophylaxis and treatment guidelines

The mainstay of treatment/prophylaxis in all hemophilia patients is to replace the deficient factor in the form of concentrate from plasma, recombinant factors, or extended half-life recombinant products. A newly introduced prophylactic option in hemophilia A is emicizumab, a humanized monoclonal antibody that stabilizes both FIXa and FX, substituting the role of FVIII [13-15].

Desmopressin (DPVV) is used in mild-to-moderate bleeding of hemophilia A; it does not have any role in hemophilia B [16]. DPVV releases subendothelial von Willebrand factor (VWF), and VWF subsequently stabilizes FVIII in plasma, increasing its half-life. Fresh frozen plasma is no longer used in hemophilia patients because of volume overload and viral elimination concerns.

The National Hemophilia Foundation has recommended the primary prophylaxis from age 1 or 2 to adolescence in patients having factor activity less than 1% and maintaining the activity above 2%. Secondary prophylaxis is indicated in all patients after hemarthrosis episodes and aimed to prevent long-term complications from recurrent bleeding in the joint [17]. For prophylaxis, 25 to 40 IUs /kg (body weight) of FVIII and FIX are administered thrice/week for hemophilia A and twice a week for hemophilia B, respectively (Malmo protocol). Long-lasting recombinant products allow once a week or once every two-week use. Inhibitor development with long-term use of replacement factors can significantly reduce the effect of exogenous factors; emicizumab does not have inhibitor issues.

It is advised that factor activity should be restored according to the medical situation (Table 2) [18].

**Table 2:** Recommended factor activity levels in acute conditions *Check factor activity 10-15 minutes after the first dose, and in major bleed, check levels 4-6 hours for FVIII and 8-12 hours for FIX. Usual loading dose for achieving 80-100% factor activity (in non-inhibitor person) is 50 IU/kg of factor VIII in hemophilia A and 100-120 IU/kg of FIX in hemophilia B. FVIII, factor VIII; FIX, factor IX

Conditions	
Minor hemorrhages/epistaxis	Can be treated with local pressure, Icing, or anti-fibrinolytics as appropriate [13]
Hemarthrosis/severe hematoma	At least 50% or above
Major bleeds/life-threatening bleeding such as large retroperitoneal hemorrhage or intracranial hemorrhages	Up to 80%-100% and continue to maintain above 50% for 10-14 days*

The following formulas [19] can be used for the replacement of FVIII and FIX in acute medical situations:

Dose (units) of FVIII = (desired factor level activity - baseline factor level activity) x (patient weight [kg]/2) 

Dose (units) of FIX = (desired factor level activity - baseline factor level activity) x patient weight (kg) x 1.2

The preceding formulas are for the initial dosing, and the maintenance dose is usually one half of the initial dose given after 12 hours of initial dose in hemophilia A (FVIII) and given after 24 hours in hemophilia B (FIX).

Other medications used in acute management are antifibrinolytics (tranexamic acid or ε-aminocaproic acid) [20]. Along with prophylaxis and acute management of bleeding, long-term management includes joint strengthening exercises and the prevention and treatment of joint deformities.

Pregnancy in female carriers

The bleeding profile usually improves in pregnancy as the clotting factor levels increase during pregnancy. FVIII levels rise, but there is no effect on FIX levels. Overall, there is no increased risk of miscarriages in hemophilia. Any female from a hemophilic family, exhibiting bleeding abnormalities, or wants to conceive should be recommended for genetic testing. If she comes out as a disease carrier, she should be counseled about it [21,22] and the following is recommended:

1) Risks of bleeding during pregnancy and her carrier status should be documented.

2) Risks and chances of transmission of disease to newborn, as carrier females have a 50% probability of inheriting diseased to children.

3) The outcome of inheriting the diseased X chromosome in a male and female child, treatment of a possible diseased child, and cost.

4) Prenatal fetal sex determination is advised, although it does not reveal the fetus's diseased status but provides valuable information for further management. If the fetus is female, prenatal diagnosis with chorionic villous sampling (CVS) or amniocentesis is not necessary as even if the female is a carrier, there is a little risk of bleeding abnormalities. If the fetus is male, prenatal diagnosis should be offered with CVS and amniocentesis.

5) Mother should be counseled about the risks and benefits of prenatal diagnostic testing. Amniocentesis carries about a 1% risk of miscarriage.

6) Vacuumed extraction and forceps delivery should be avoided.

7) Circumcision should not be performed in male babies born to hemophiliac or carrier mothers until the newborn's disease has been excluded.

Although a physician can offer genetic testing in a female carrier and prenatal testing for a baby, the final decision regarding testing relies on the mother/family.

## Conclusions

Classical homozygous hemophilia is very rarely seen in females. It is mostly in those communities where consanguinity is common, such as the subcontinent. Despite the fact, the manifestations of hemophilia are common in female carriers. This needs prompt attention and diagnosis of this disease to decrease short- and long-term complications and to improve hemophilia patients' quality of life.

## Appendices

**Table 3:** Useful resources for hemophilia patients and physicians

Resources	
World Federation of Hemophilia	https://www.wfh.org/en/home
The National Hemophilia Foundation	https://www.hemophilia.org/
LA Kelley Communications Inc.	https://www.kelleycom.com/
Centers for Disease Control and Prevention	https://www.cdc.gov/dotw/hemophilia/index.html
Hemophilia Federation of America	https://www.hemophiliafed.org/
